# Supplementary material for: KPNA3 regulates histone locus body formation by modulating condensation and nuclear import of NPAT
Source: J Cell Biol. 2024 Dec 2;224(1):e202401036. doi: 10.1083/jcb.202401036 (PMC11613458; doi:10.1083/jcb.202401036)
Supplement: SourceData F1 — is the source file for Fig. 1. [file jcb_202401036_sourcedataf1.pdf]

SourceData F1

1D

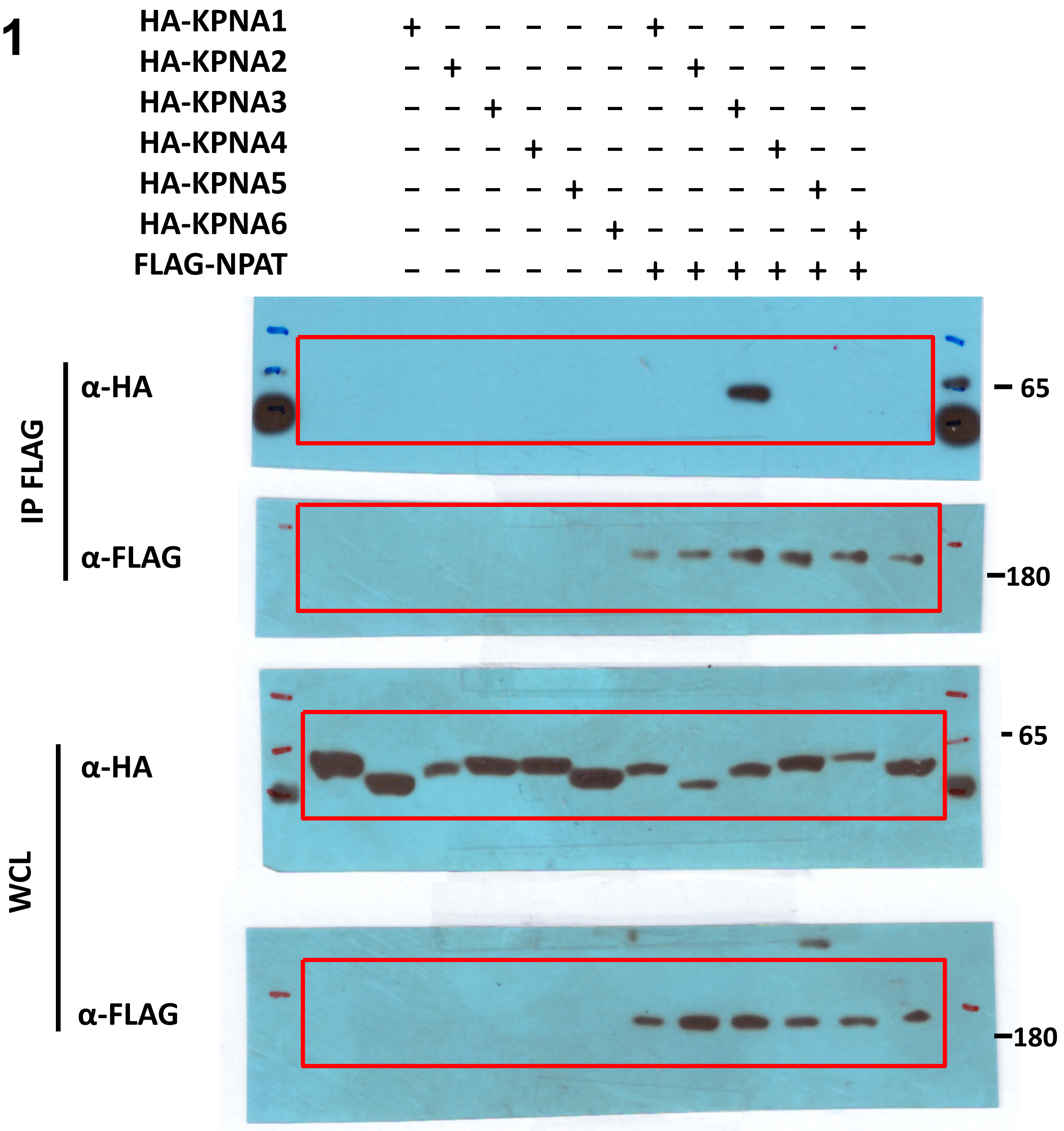

1F

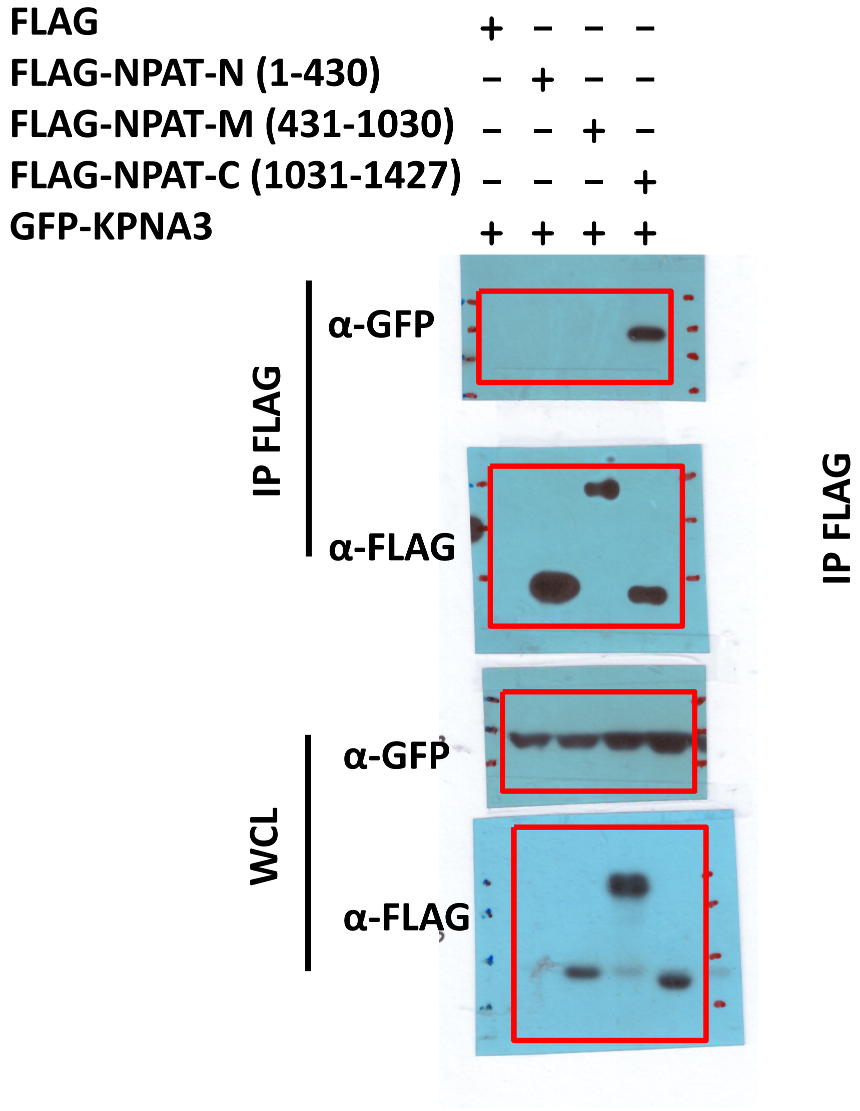
FLAG + − − −
FLAG-NPAT-N (1-430) − + − −
FLAG-NPAT-M (431-1030) − − + −
FLAG-NPAT-C (1031-1427) − − − +
GFP-KPNA3 + + + +
IP FLAG
α-GFP
α-FLAG
WCL
α-GFP
α-FLAG
IP FLAG

1I

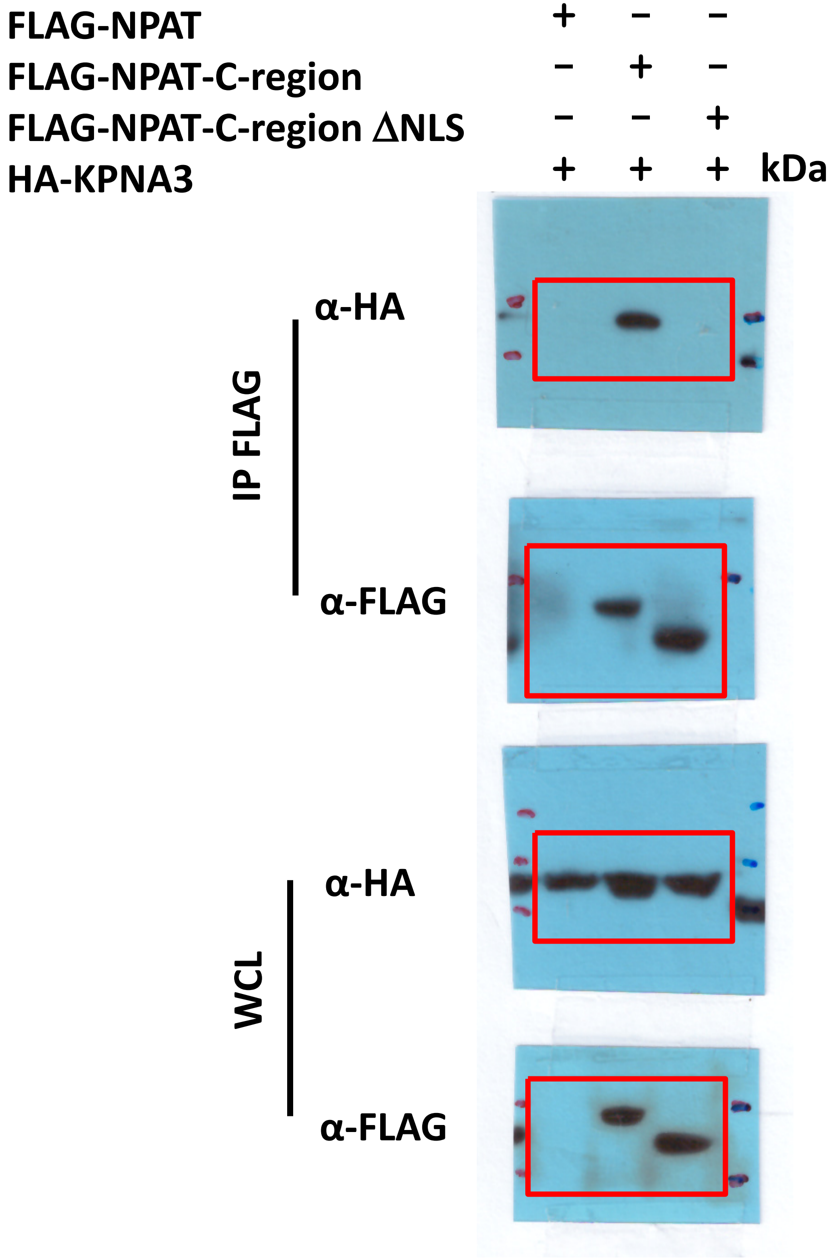
FLAG-NPAT + − −
FLAG-NPAT-C-region − + −
FLAG-NPAT-C-region ΔNLS − − +
HA-KPNA3 + + +
kDa
IP FLAG
α-HA
α-FLAG
WCL
α-HA
α-FLAG
