# Supplementary figures and images for: KPNA3 regulates histone locus body formation by modulating condensation and nuclear import of NPAT
Source: J Cell Biol. 2024 Dec 2;224(1):e202401036. doi: 10.1083/jcb.202401036 (PMC11613458; doi:10.1083/jcb.202401036)

2A

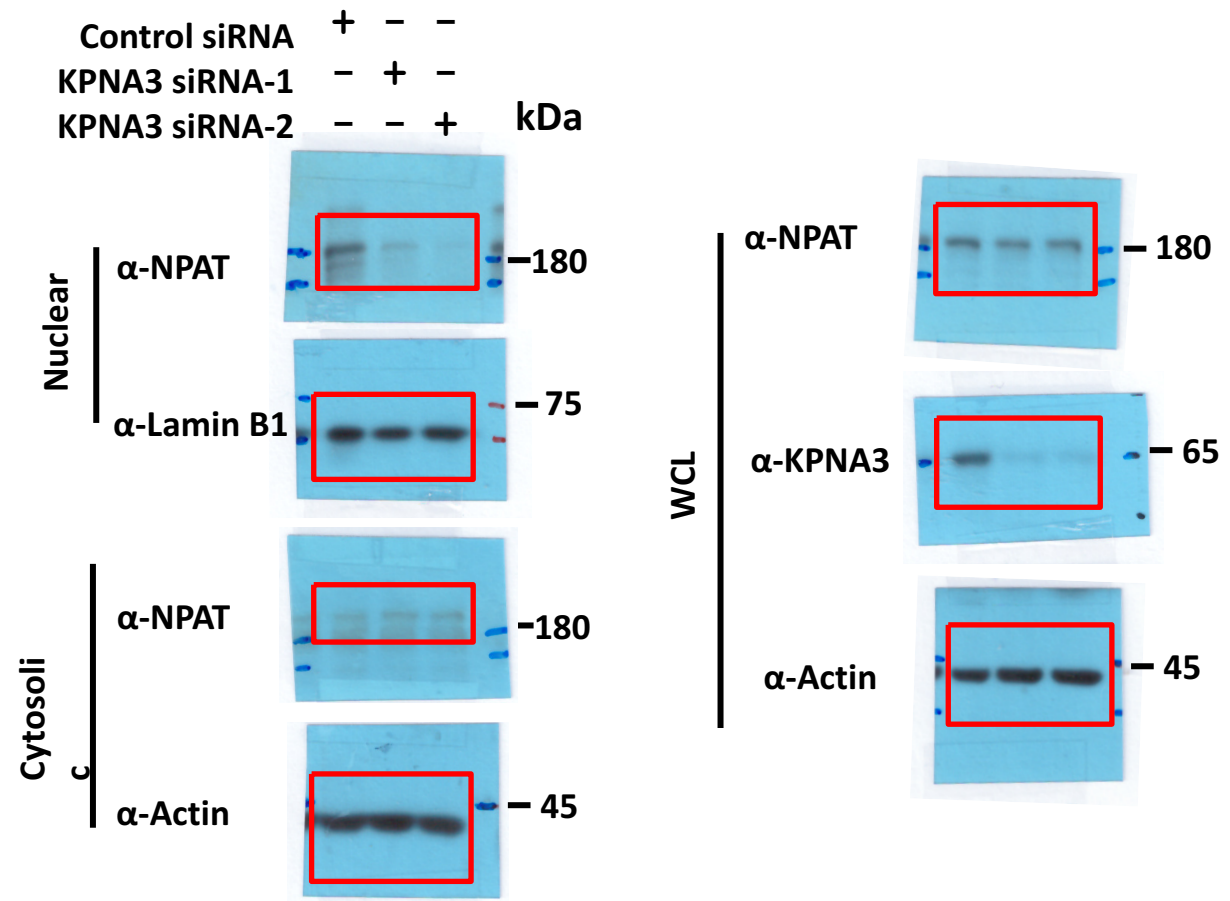

WCL

α-NPAT

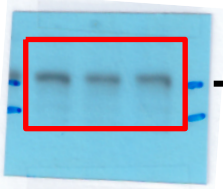

-180

α-KPNA3

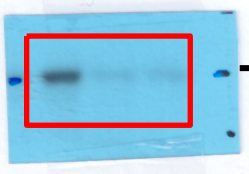

-65

α-Actin

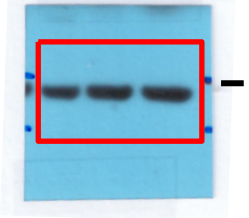

-45

2C

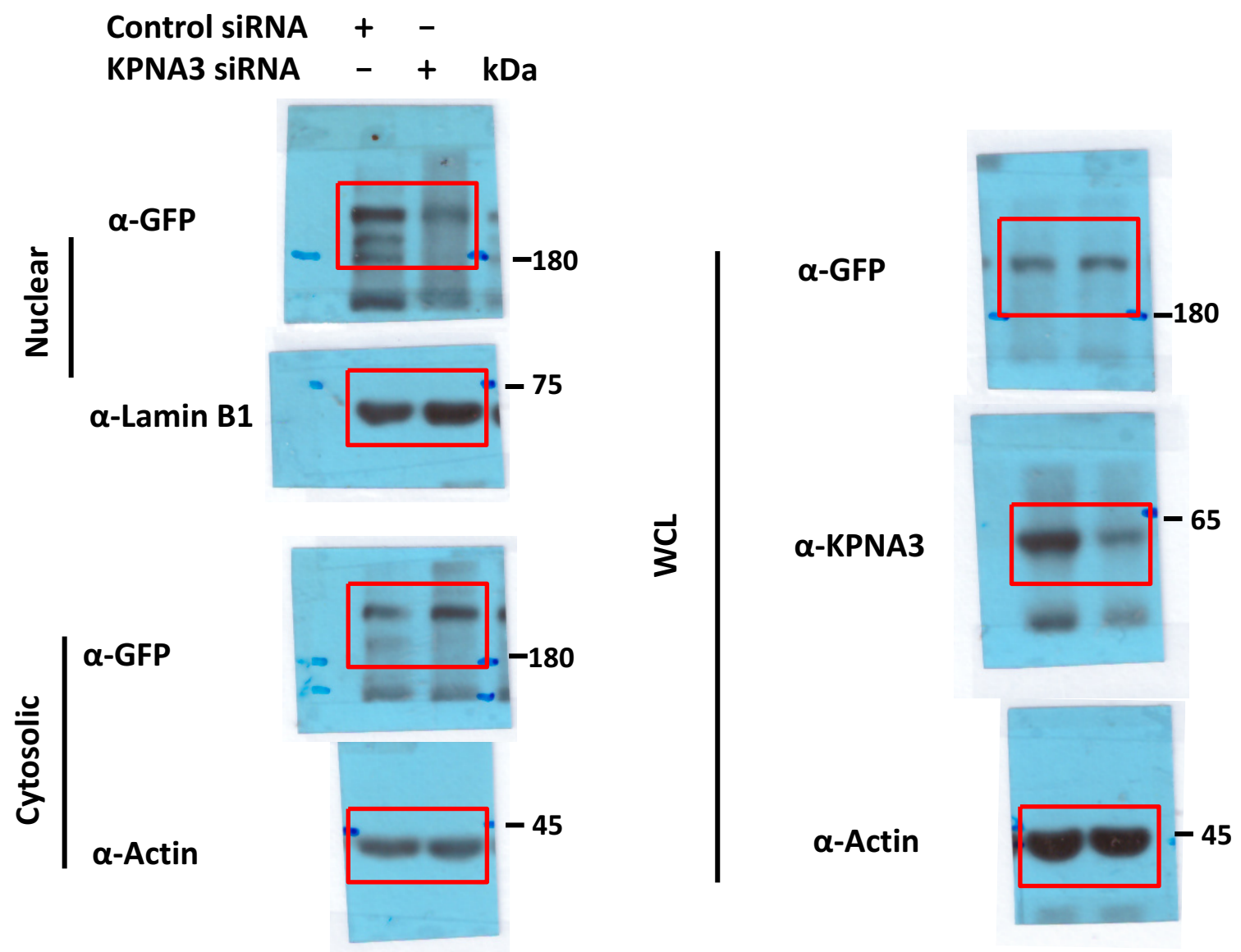

Supplement: SourceData F2 — is the source file for Fig. 2. [file jcb_202401036_sourcedataf2.pdf]

5C

|                     |   |   |     |
|---------------------|---|---|-----|
| FLAG                | + | - | -   |
| FLAG-KPNA5          | - | + | -   |
| FLAG-KPNA5-3 hybrid | - | - | +   |
| GFP-NPAT C region   | + | + | +   |
|                     |   |   | kDa |

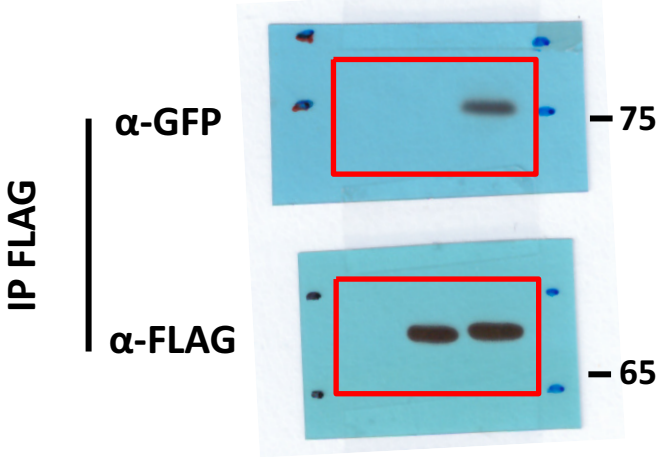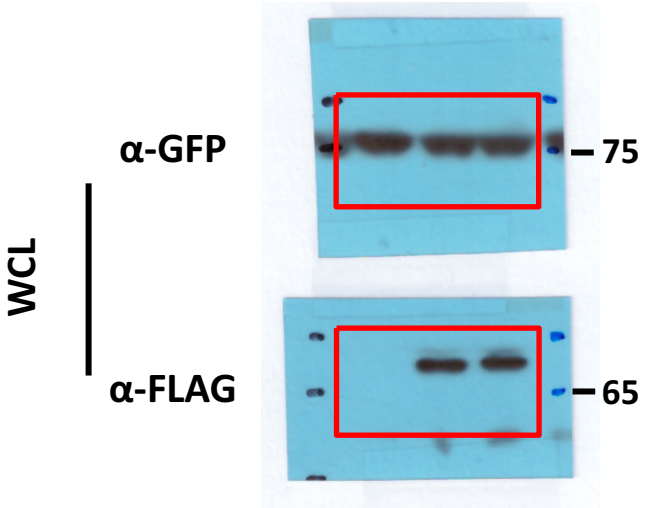

5F

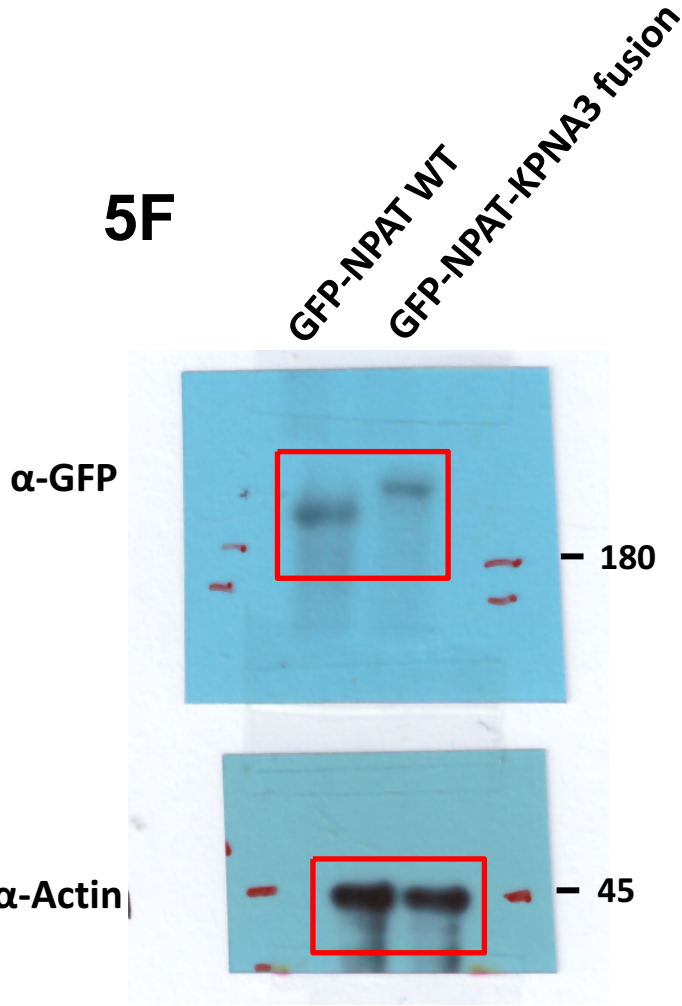

Supplement: SourceData F5 — is the source file for Fig. 5. [file jcb_202401036_sourcedataf5.pdf]

6A

|                                |        |   |   |   |
|--------------------------------|--------|---|---|---|
|                                | FLAG + |   |   |   |
| FLAG-NPAT N-region (1-430)     | +      |   |   |   |
| FLAG-NPAT M-region (431-1030)  |        | + |   |   |
| FLAG-NPAT C-region (1031-1427) |        |   | + |   |
| GFP-C-region (1031-1427)       | +      | + | + | + |

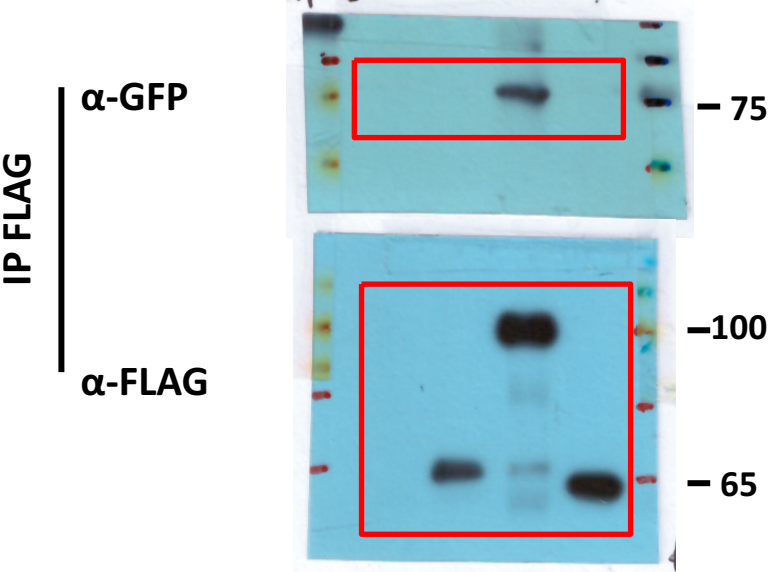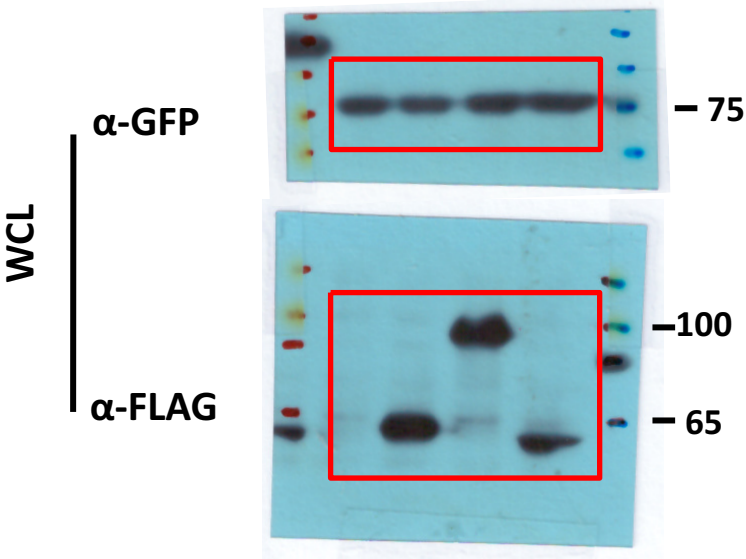

6C

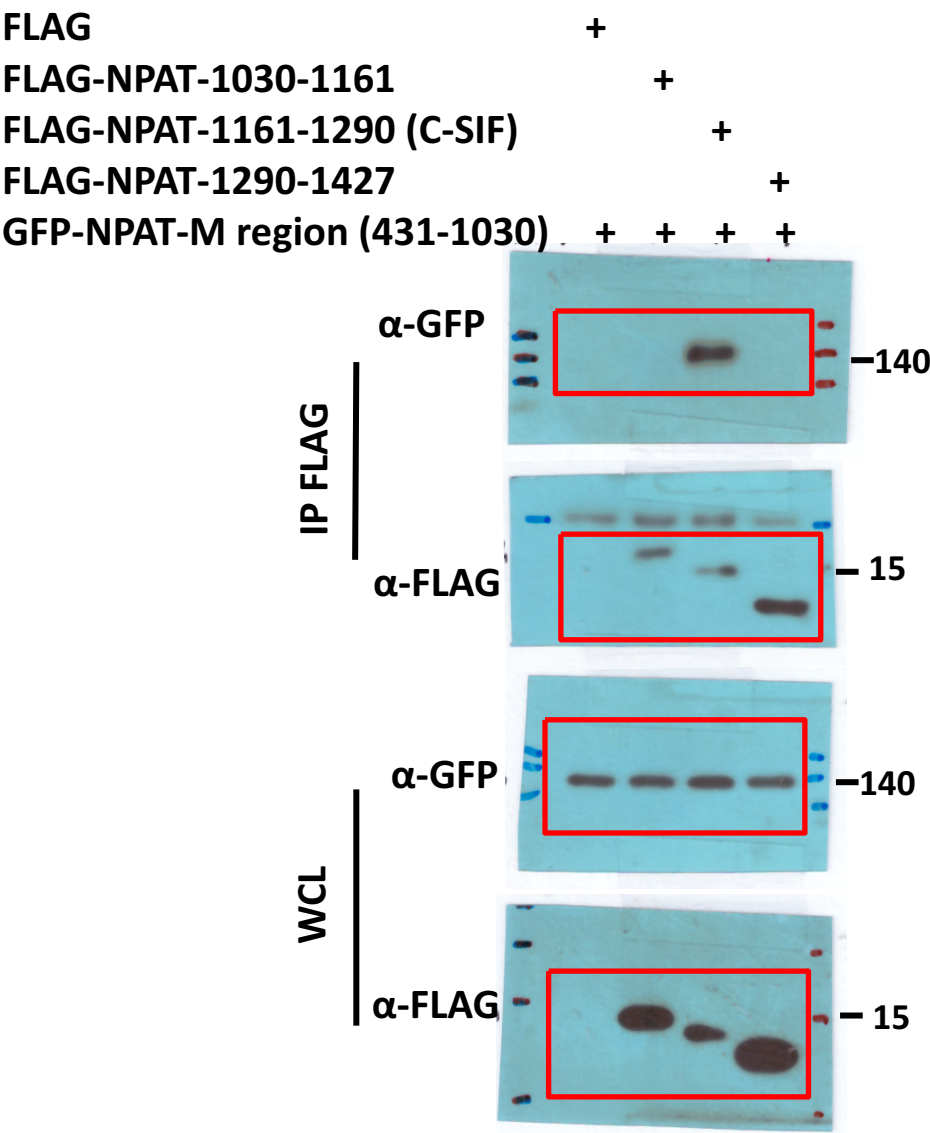

6D

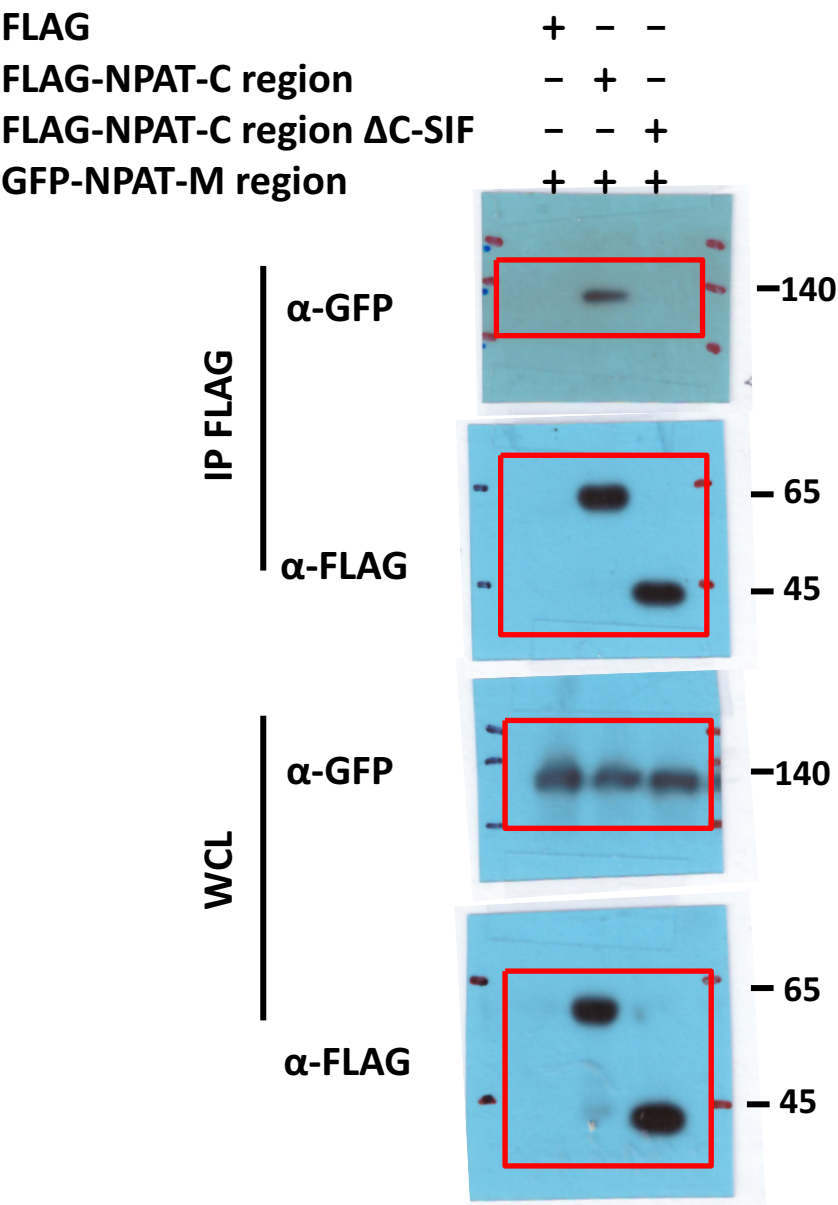

6F

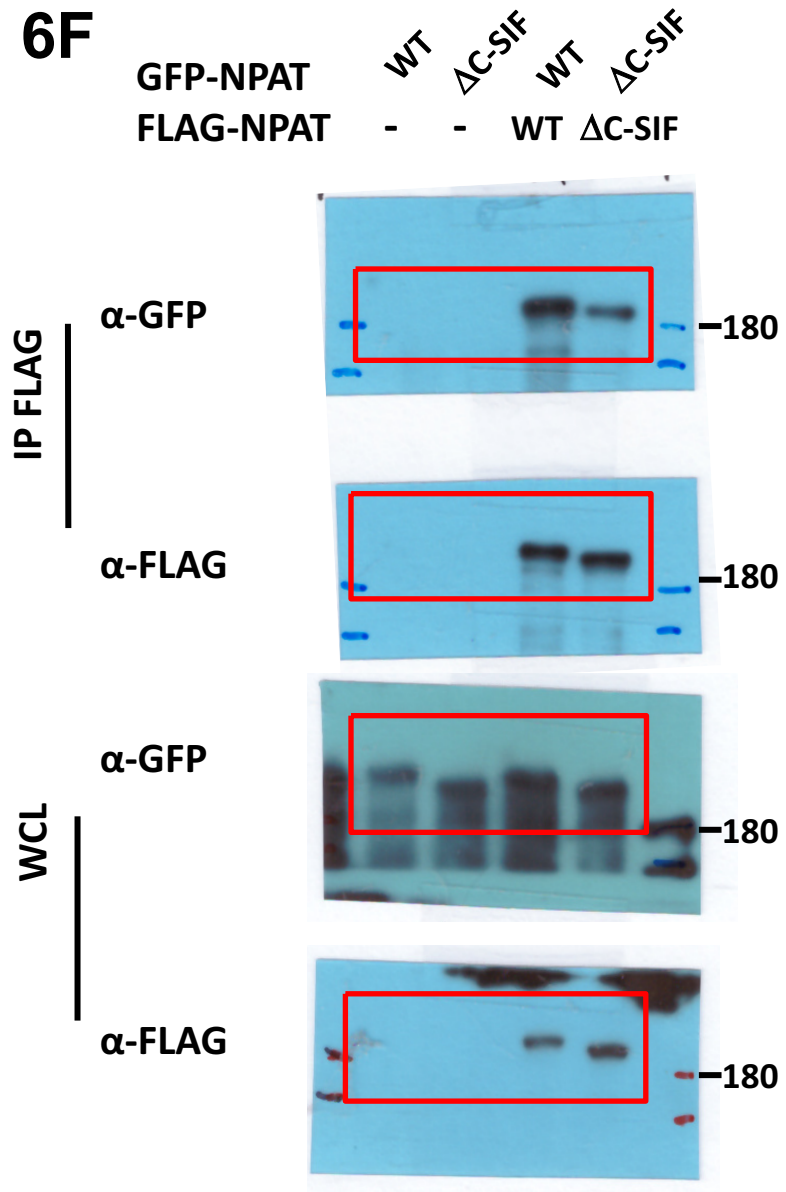

6H

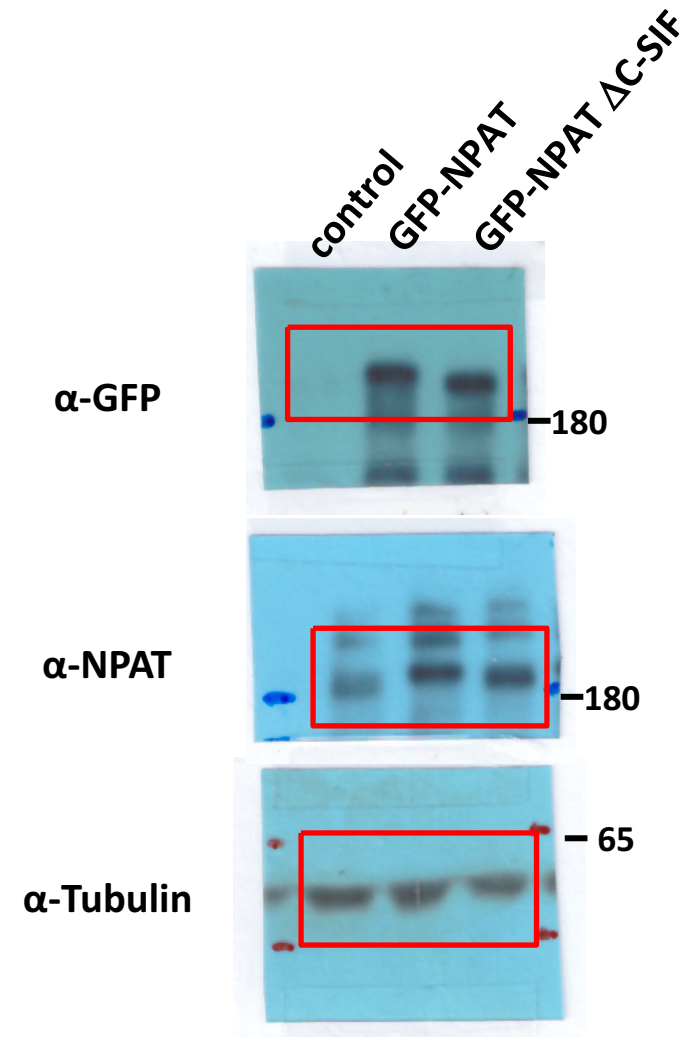

6J

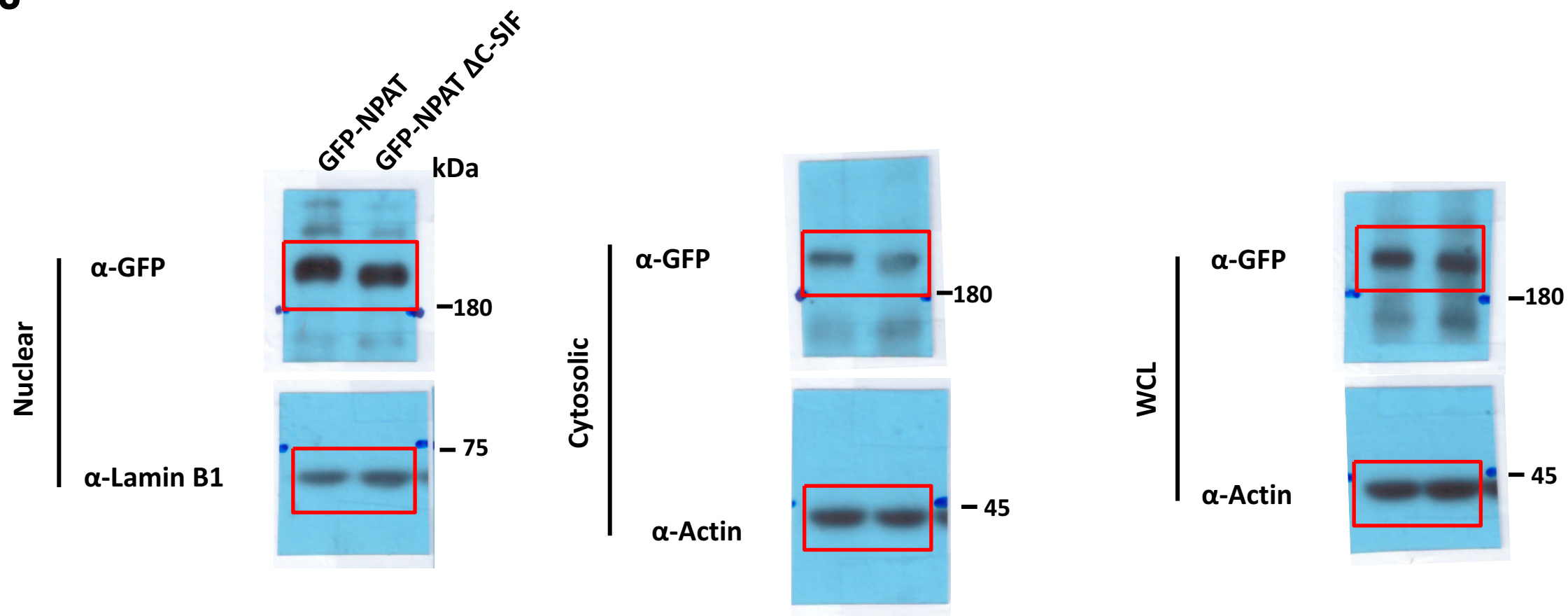

6J

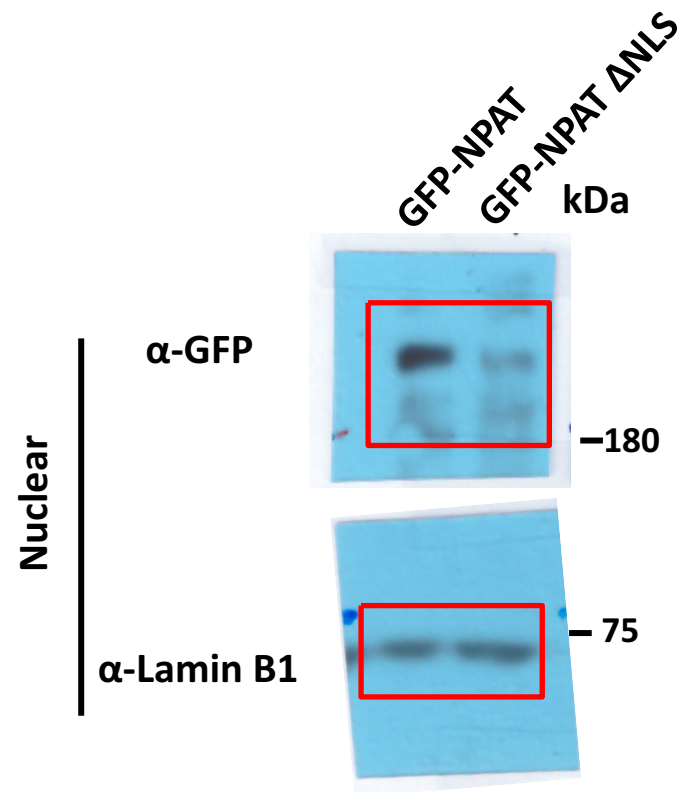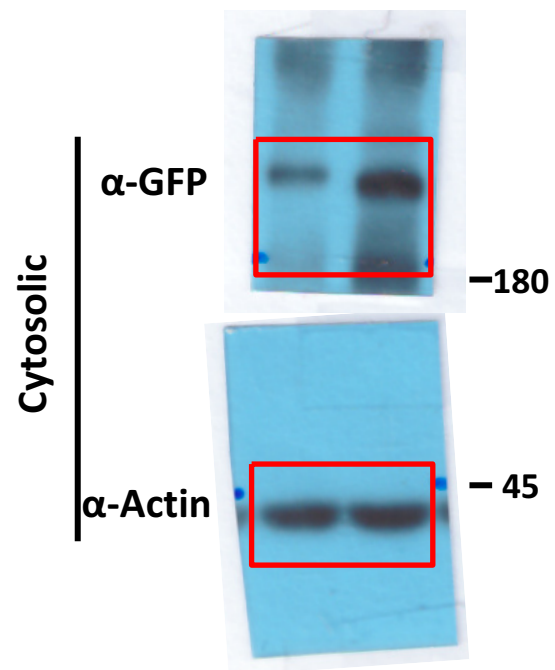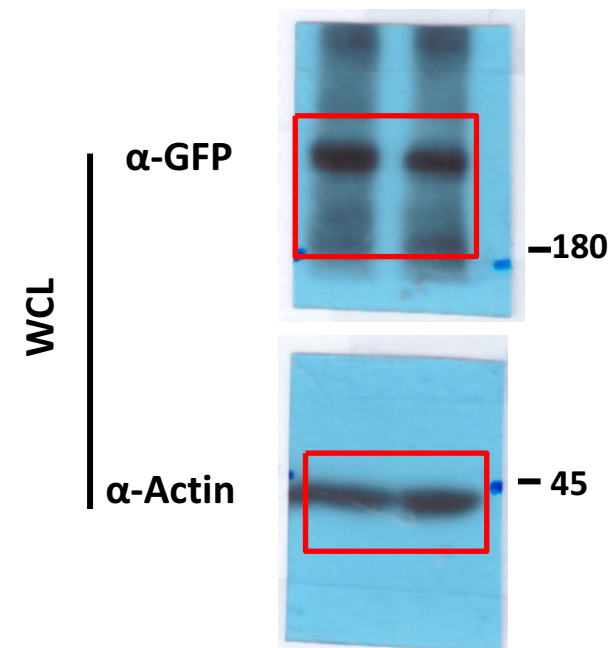

Supplement: SourceData F6 — is the source file for Fig. 6. [file jcb_202401036_sourcedataf6.pdf]

S1A

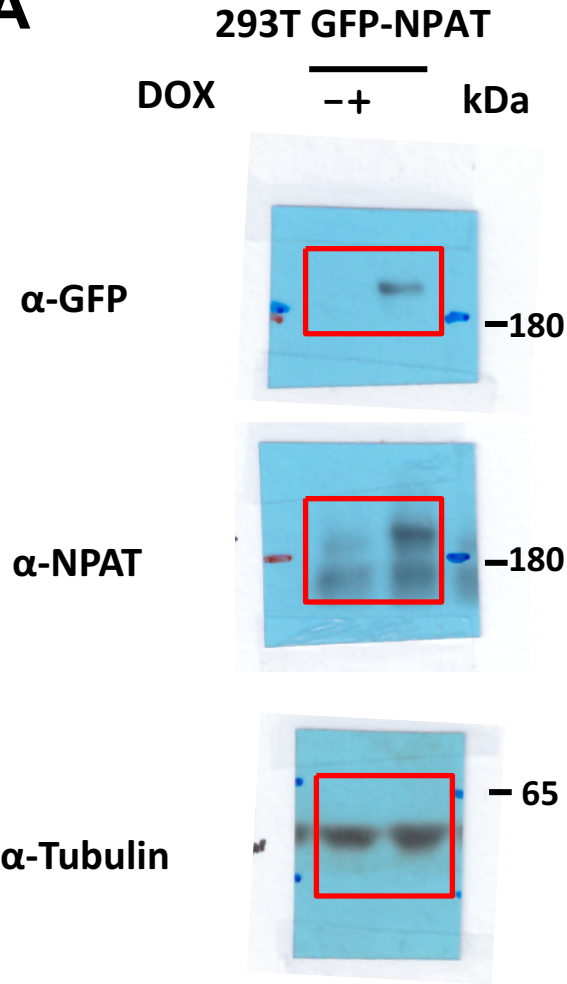

Supplement: SourceData FS1 — is the source file for Fig. S1. [file jcb_202401036_sourcedatafs1.pdf]

S2A

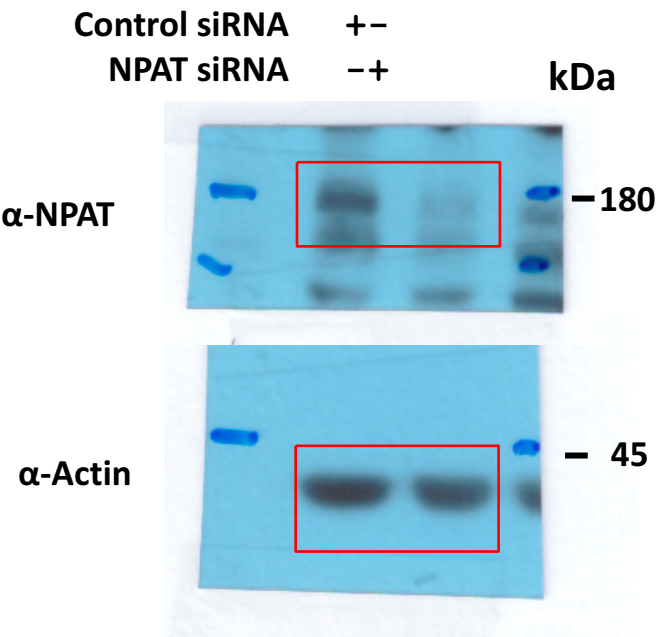

S2B

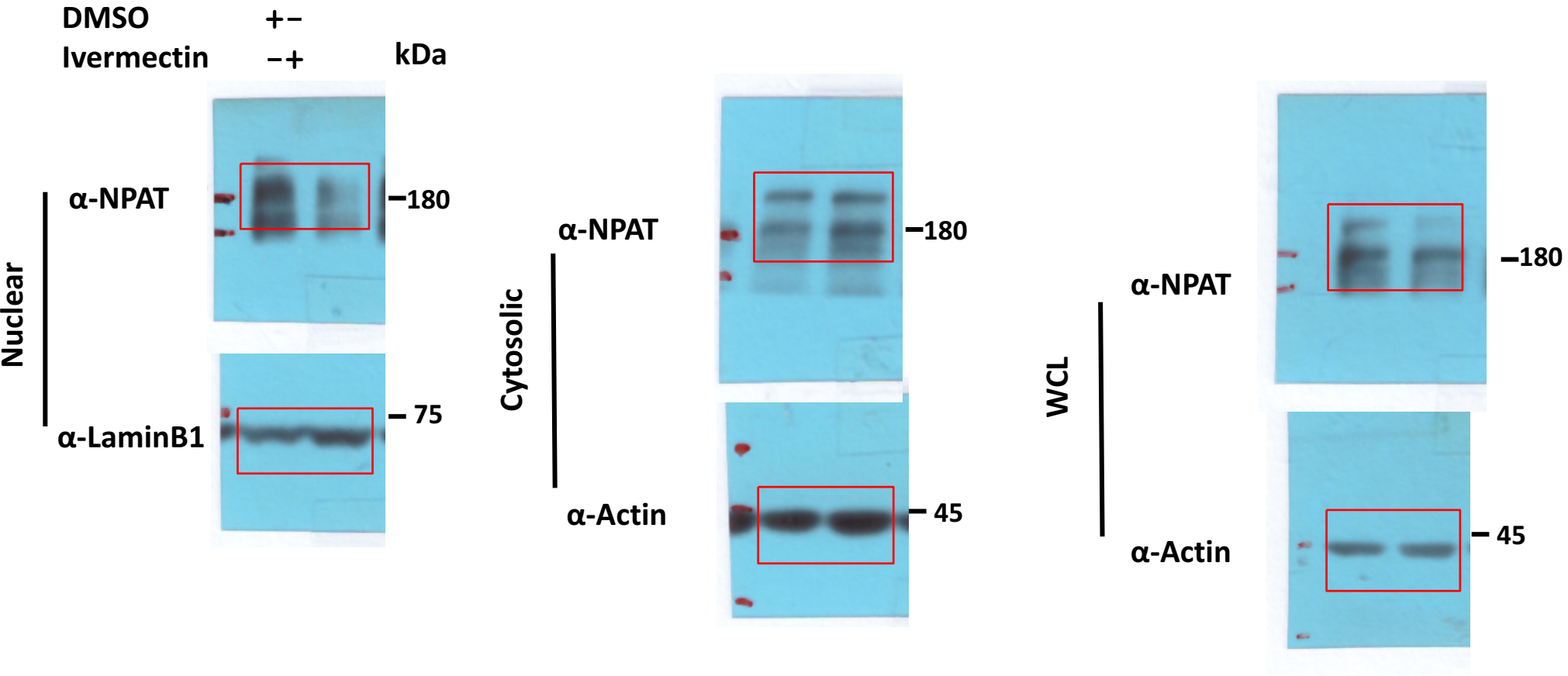

# S2D

|               |    |     |
|---------------|----|-----|
| Control siRNA | +- |     |
| KPNA3 siRNA   | -+ | kDa |

$\alpha$ -KPNA3

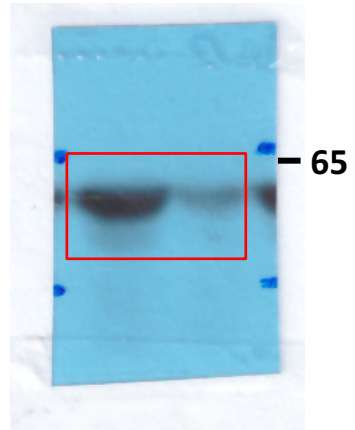

$\alpha$ -Actin

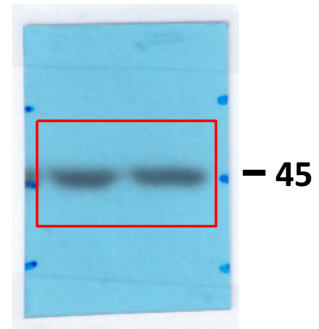

Supplement: SourceData FS2 — is the source file for Fig. S2. [file jcb_202401036_sourcedatafs2.pdf]

# SourceDataFS3

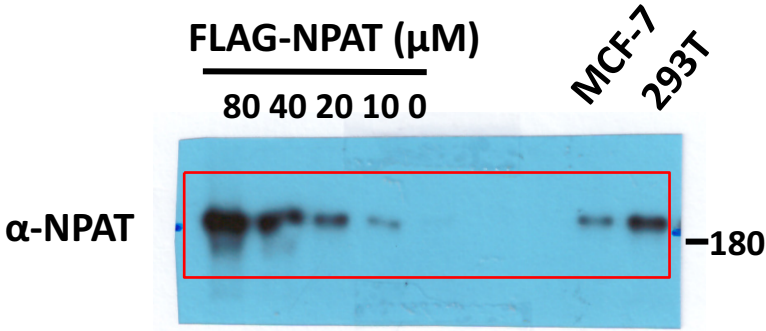

Supplement: SourceData FS3 — is the source file for Fig. S3. [file jcb_202401036_sourcedatafs3.pdf]
