# Supplementary material for: KPNA3 regulates histone locus body formation by modulating condensation and nuclear import of NPAT
Source: J Cell Biol. 2024 Dec 2;224(1):e202401036. doi: 10.1083/jcb.202401036 (PMC11613458; doi:10.1083/jcb.202401036)
Supplement: SourceData F4 — is the source file for Fig. 4. [file jcb_202401036_sourcedataf4.pdf]

SourceDataF4

4B

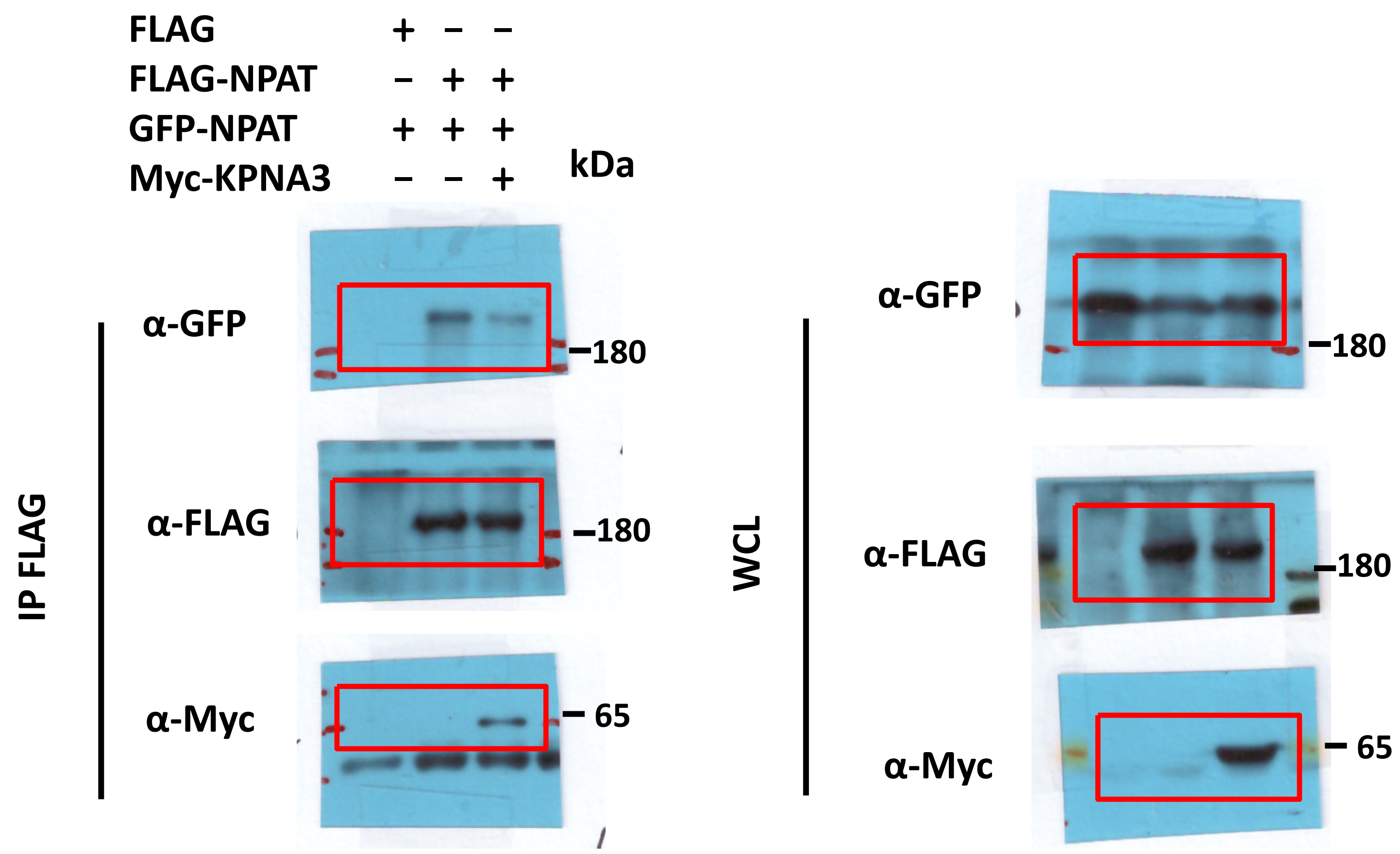

4D

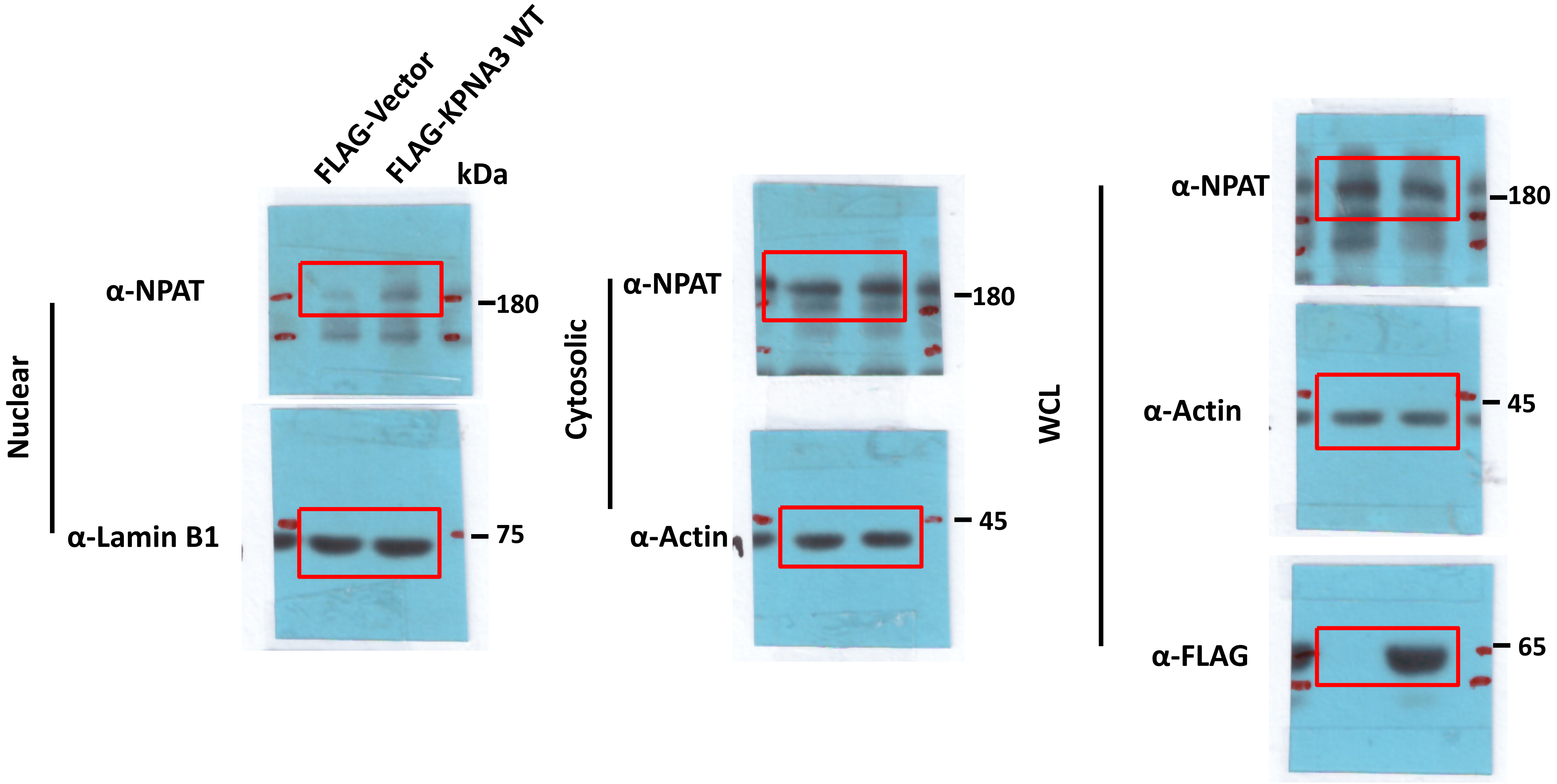
FLAG-Vector
FLAG-KPNA3 WT
kDa
Nuclear
α-NPAT
180
α-Lamin B1
75
Cytosolic
α-NPAT
180
α-Actin
45
WCL
α-NPAT
180
α-Actin
45
α-FLAG
65

4H

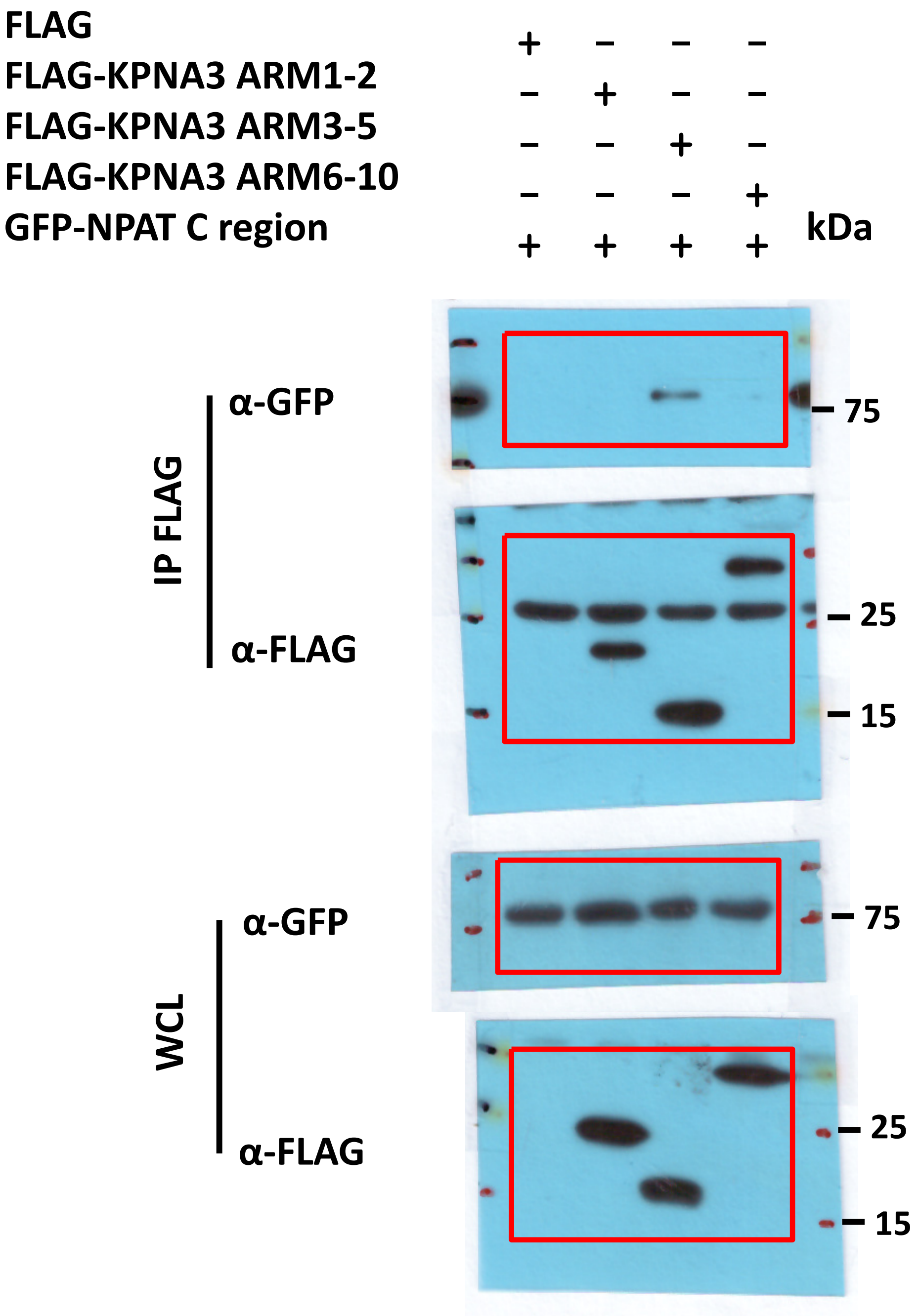
FLAG
FLAG-KPNA3 ARM1-2
FLAG-KPNA3 ARM3-5
FLAG-KPNA3 ARM6-10
GFP-NPAT C region
+ – – –
– + – –
– – + –
– – – +
+ + + +
kDa
IP FLAG
α-GFP
α-FLAG
WCL
α-GFP
α-FLAG
75
25
15
75
25
15

4J

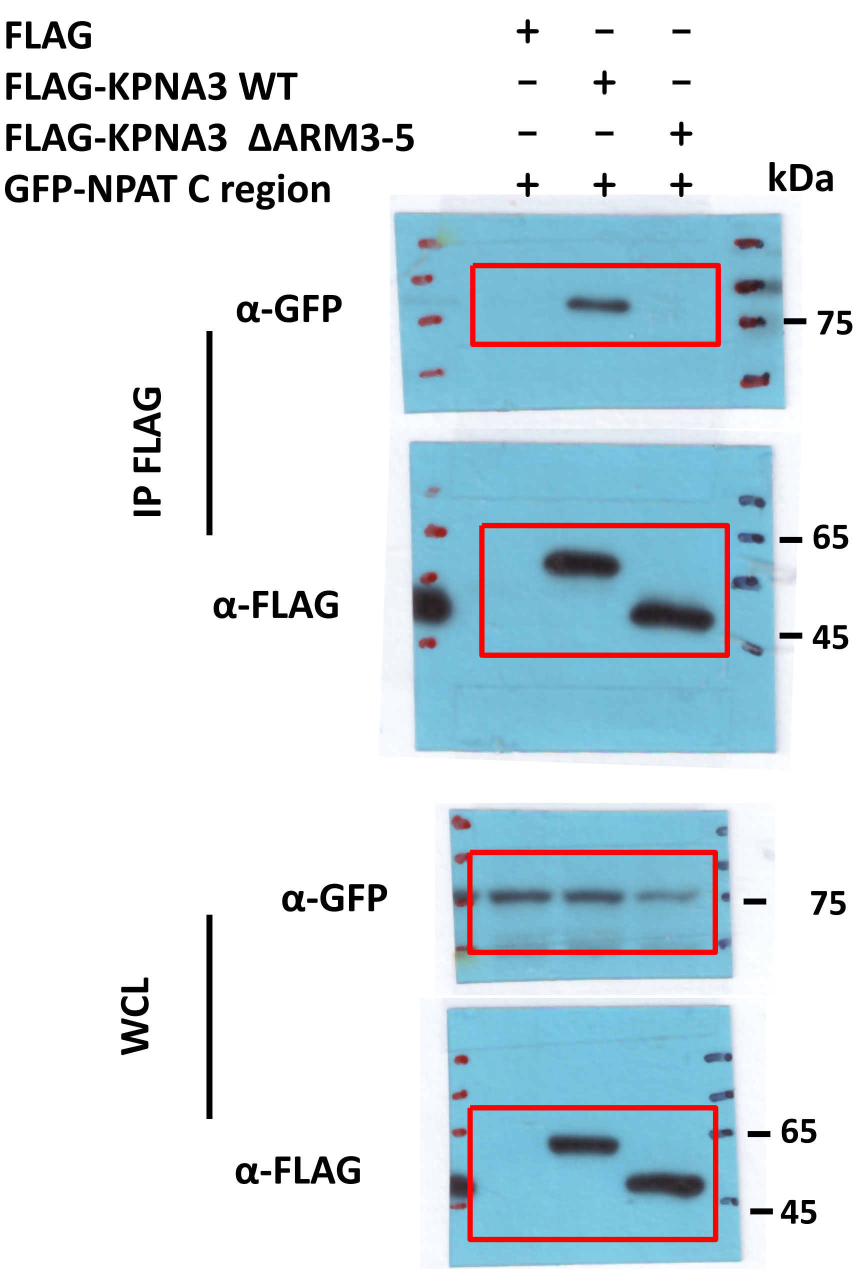
FLAG
FLAG-KPNA3 WT
FLAG-KPNA3 ΔARM3-5
GFP-NPAT C region
+ – –
– + –
– – +
+ + +
kDa
IP FLAG
α-GFP
α-FLAG
WCL
α-GFP
α-FLAG
75
65
45
75
65
45
