# Supplementary material for: KPNA3 regulates histone locus body formation by modulating condensation and nuclear import of NPAT
Source: J Cell Biol. 2024 Dec 2;224(1):e202401036. doi: 10.1083/jcb.202401036 (PMC11613458; doi:10.1083/jcb.202401036)
Supplement: SourceData F7 — is the source file for Fig. 7. [file jcb_202401036_sourcedataf7.pdf]

SourceDataF7

7A

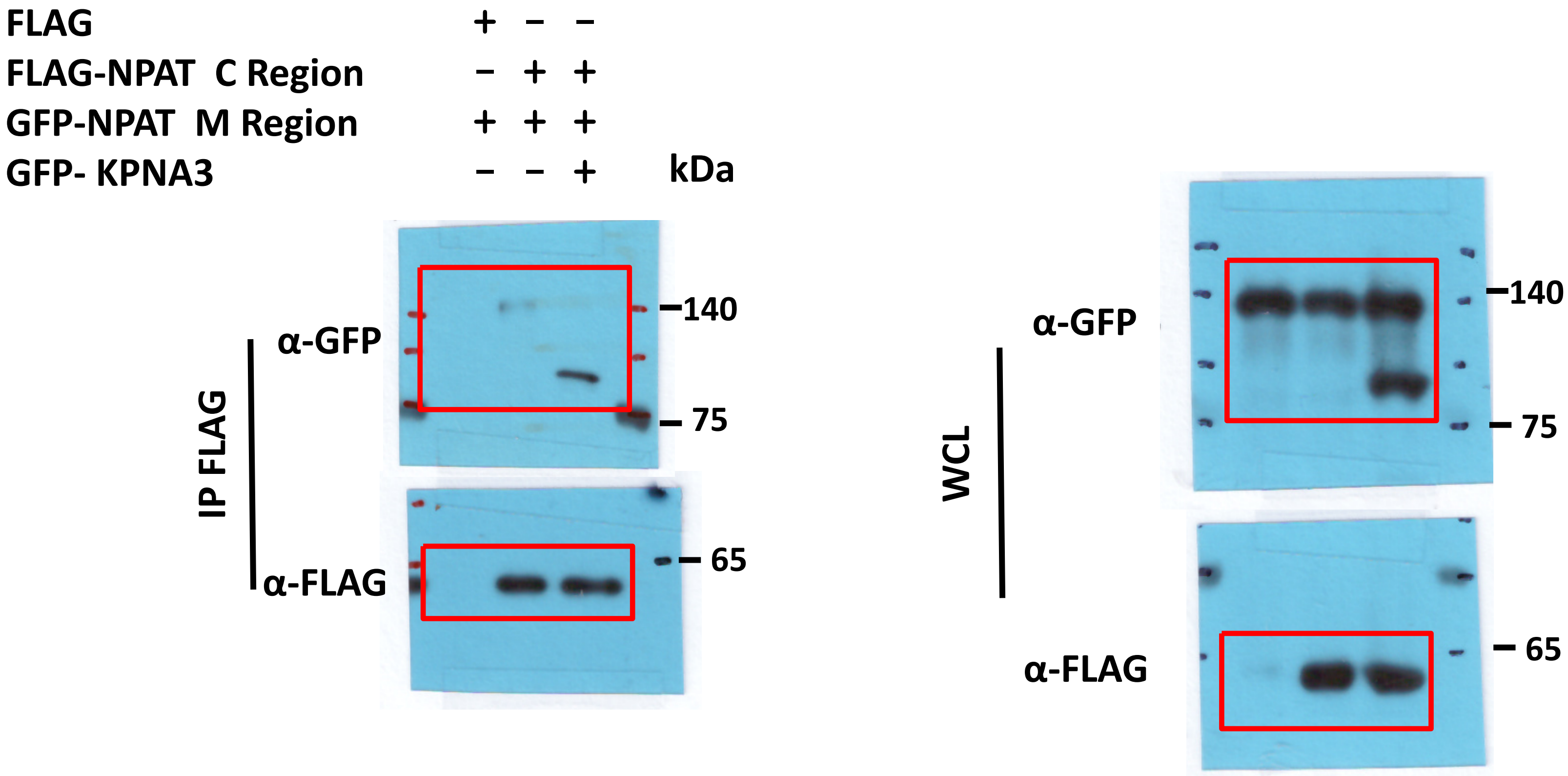

7B

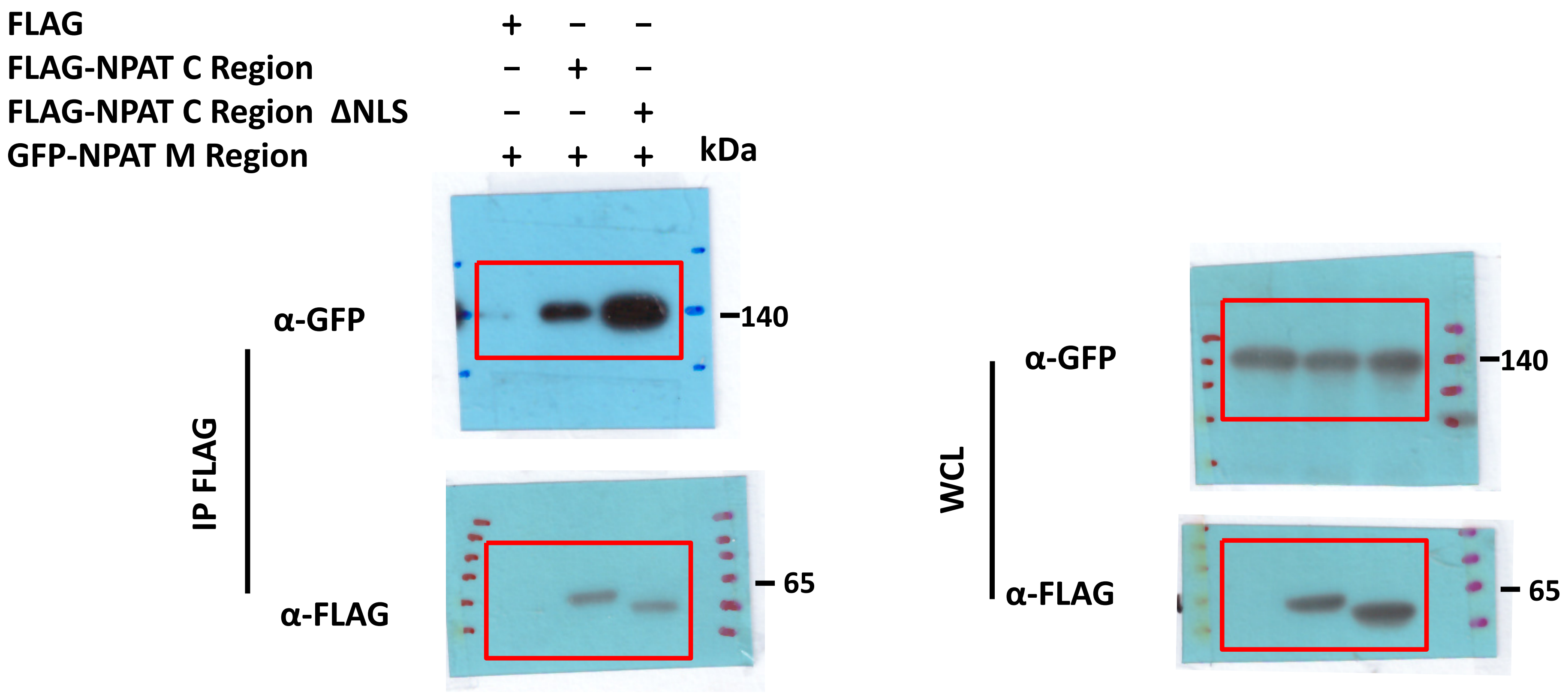
FLAG
FLAG-NPAT C Region
FLAG-NPAT C Region ΔNLS
GFP-NPAT M Region
kDa
α-GFP
140
IP FLAG
α-FLAG
65
WCL
α-GFP
140
α-FLAG
65

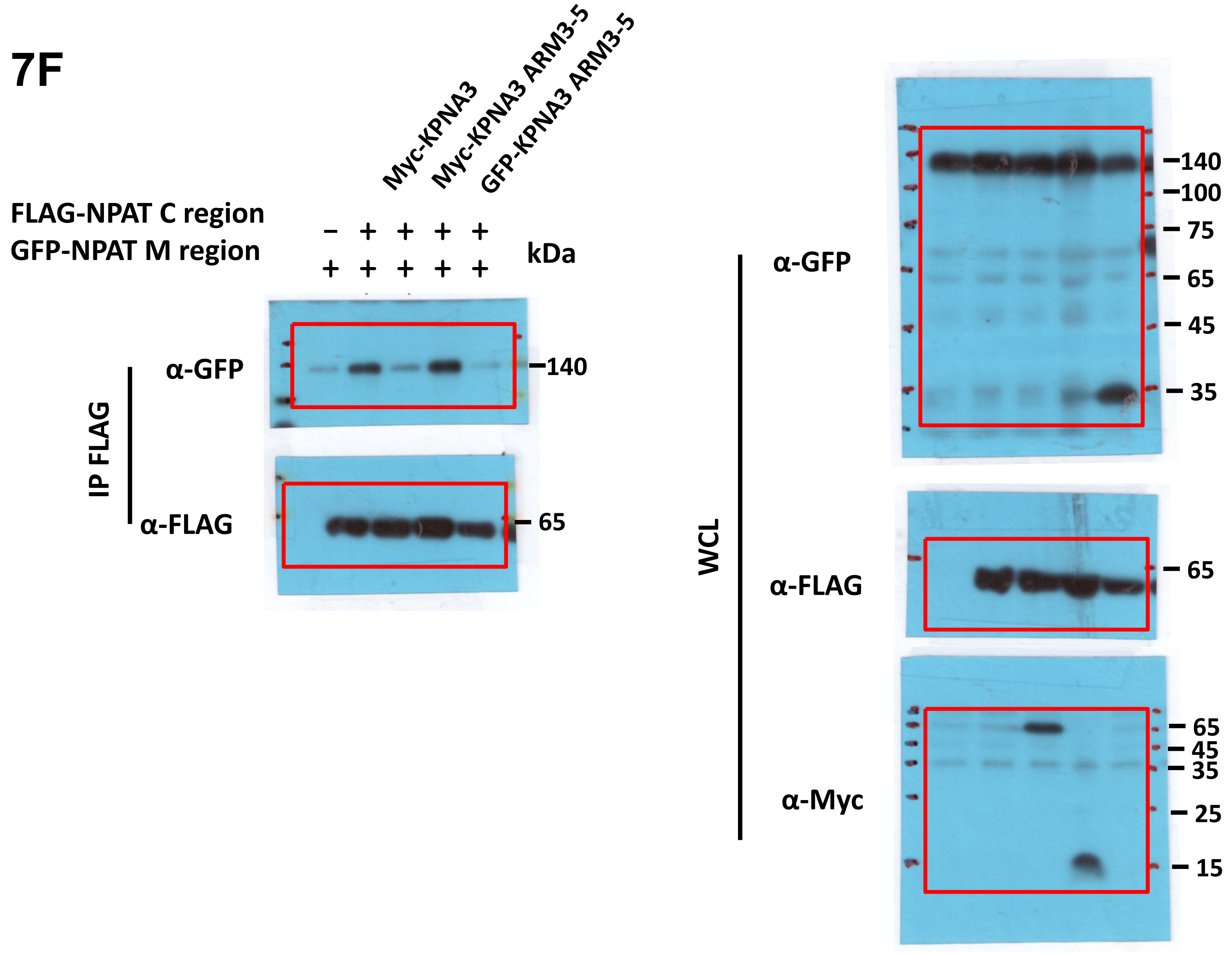
7F
Myc-KPNA3
Myc-KPNA3 ARM3-5
GFP-KPNA3 ARM3-5
FLAG-NPAT C region
GFP-NPAT M region
– + + + +
+ + + + +
kDa
IP FLAG
α-GFP
140
α-FLAG
65
WCL
α-GFP
140
100
75
65
45
35
α-FLAG
65
α-Myc
65
45
35
25
15

7I

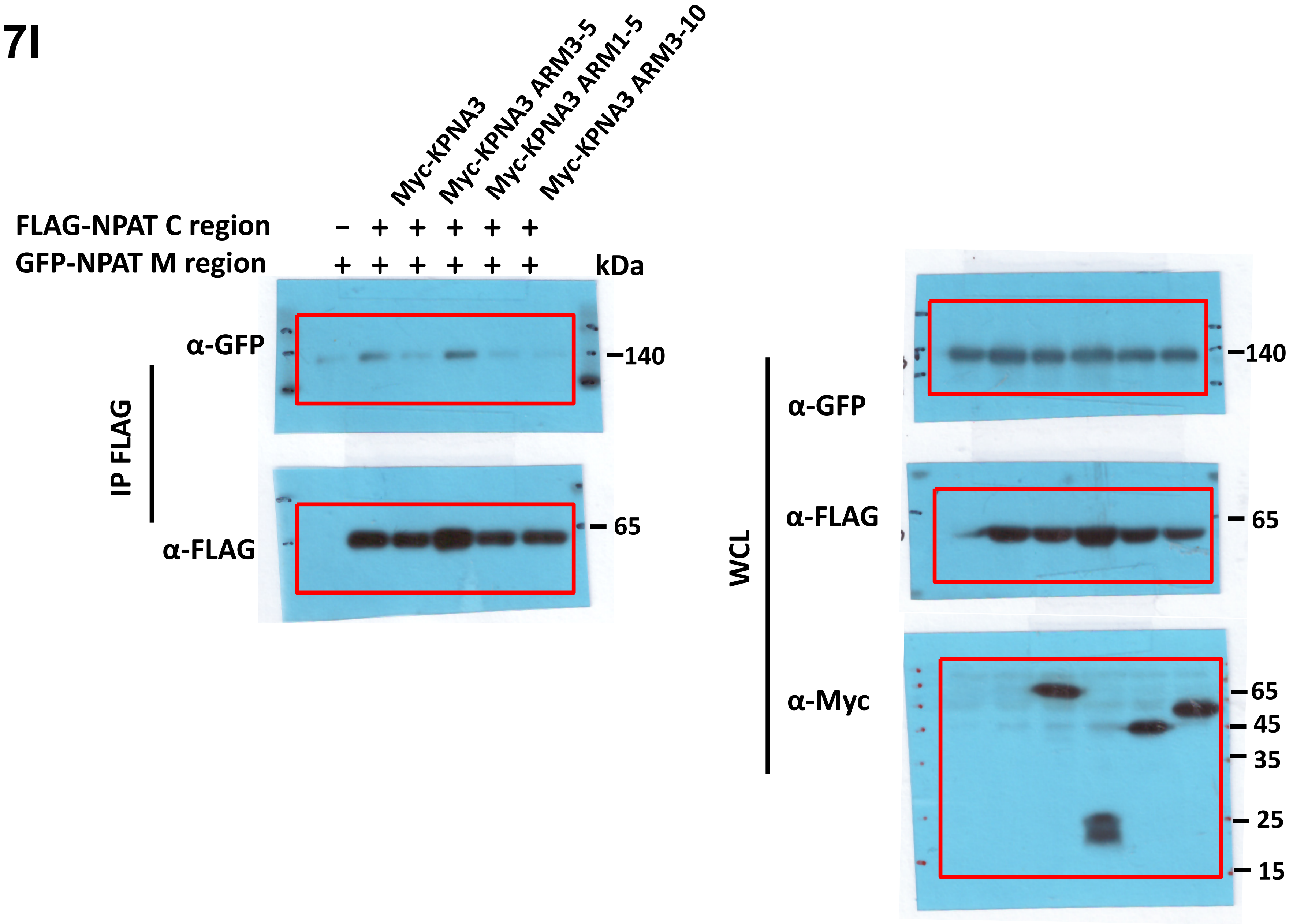

Myc-KPNA3
Myc-KPNA3 ARM3-5
Myc-KPNA3 ARM1-5
Myc-KPNA3 ARM3-10
FLAG-NPAT C region – + + + + +
GFP-NPAT M region + + + + + +
kDa
α-GFP
140
IP FLAG
α-FLAG
65
WCL
α-GFP
140
α-FLAG
65
α-Myc
65
45
35
25
15
